# HDSS Profile: The South East Asia Community Observatory Health and Demographic Surveillance System (SEACO HDSS)

**DOI:** 10.1093/ije/dyx113

**Published:** 2017-07-26

**Authors:** Uttara Partap, Elizabeth H Young, Pascale Allotey, Ireneous N Soyiri, Nowrozy Jahan, Kridaraan Komahan, Nirmala Devarajan, Manjinder S Sandhu, Daniel D Reidpath

**Affiliations:** 1 Department of Medicine, University of Cambridge, Cambridge, UK; 2 Wellcome Trust Sanger Institute, Hinxton, UK; 3 Jeffrey Cheah School of Medicine and Health Sciences, Monash University Malaysia, Selangor, Malaysia; 4 South East Asia Community Observatory, Segamat, Malaysia; 5 Centre of Medical Informatics, Usher Institute of Population Health Sciences and Informatics, University of Edinburgh, UK

## Why was the HDSS set up?

### Background

The economic and demographic transitions under way in Asian populations have led to rapid changes in the burden of disease in the region, with large increases in cardiometabolic disease prevalence concurrent with a decreasing yet notable infectious disease burden.^1^ Comprehensive evidence from large-scale data resources based in Asian populations is required to gain a detailed understanding of this situation. Such evidence would help inform effective strategies to address the public health challenges it is anticipated to present. As a multi-ethnic middle-income South East Asian country, Malaysia provides a suitable backdrop to exploring the effect of a wide range of exposures on health and disease in populations of Malay, Chinese, Indian and indigenous (Orang Asli) ethnic origins. Its experience may be relevant to nearby countries reaching similar levels of development.

### SEACO: establishment and objectives

The South East Asia Community Observatory (SEACO) health and demographic surveillance system was established in Segamat, Malaysia, in 2011. It is operationally managed through Monash University Malaysia (MUM), and obtains core funding as a Monash technology research platform from MUM [including the Jeffrey Cheah School of Medicine and Health Sciences (JCSMHS)] and Monash University Australia (with contributions from the faculties of Medicine, Nursing and Health Sciences and of Arts). SEACO’s objective is to capture detailed longitudinal information related to health and disease among individuals and families, which can ultimately be used to improve the health experience in the local community and the wider population. It aims to achieve these objectives through: (i) the regular collection of socio-demographic and health measures among individuals; and (ii) providing a research platform for focused studies on issues related to health within the community.

## Location

**Figure 1:**
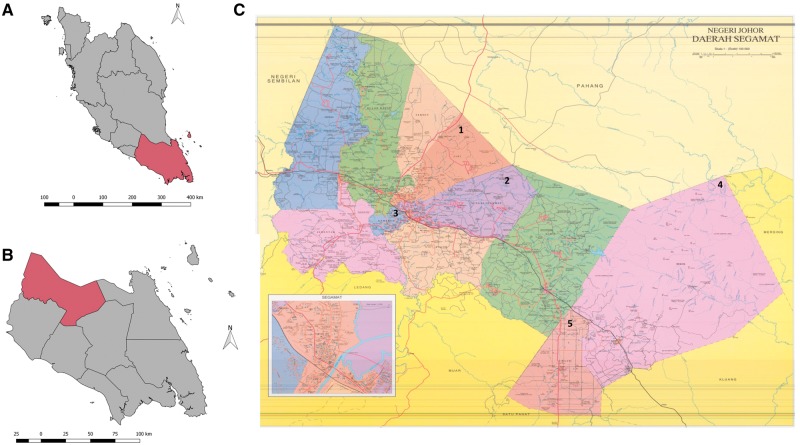
Maps of the SEACO HDSS location: (A) map of peninsular Malaysia (grey) with the state of Johor highlighted in burgundy; (B) Johor state (grey) with Segamat district highlighted in burgundy; (C) Segamat district with the five sub-districts covered by SEACO numbered: (1) Jabi (2) Sungai Segamat (3) Gemereh (in blue) (4) Bekok (5) Chaah. The map scale reference as drawn is 10 km.

SEACO is located in Segamat, the northernmost district in the southern peninsular state of Johor (approximate latitude and longitude: 2.5°N, 102.8°E). It operates in five of 11 sub-districts of Segamat, namely: Bekok, Chaah, Gemereh, Jabi and Sungai Segamat, covering a total area of approximately 1250 km^2^ (Figure 1). Segamat and these five sub-districts were selected in view of the strong pre-existing relationships between JCSMHS and the district and state health administration, essential for feasibility, sustainability and effective research translation.

The sub-districts vary in terms of size, population density, ethnic mix and level of urbanization. Segamat has a climate similar to the rest of peninsular Malaysia. Temperatures range 24–35°C throughout the year, with high humidity (70–90%) and two monsoon seasons from May to September and November to February.^2^ The area occasionally experiences flooding; the most recent floods occurred in January 2017.

Agriculture is a top private sector employer in the region, and land use in much of the rural areas comprises palm and rubber plantations.^3^ A large number of young adults migrate to larger cities, such as Kuala Lumpur or Singapore, for employment and educational opportunities. The ethnic make-up of the Segamat population according to the baseline SEACO enumeration (2012–13) was 62% Malay, 18% Chinese and 10% Indian, with 2% indigenous groups (Orang Asli) and the remainder (8%) being of other ethnicity or foreign nationals, predominantly plantation workers. This is distinct from district-, state- and national-level distributions (Table 1).^3–6^

**Table 1:** Ethnic make-up of the population covered by the SEACO HDSS, compared with district, state and national estimates

Ethnicity	Proportion of total population (%)			
	SEACO sub-districts (2012–2013)	Segamat district (2010)	Johor state (2013)	Malaysia (full) (2013)
**Malay**	62.4	52.6	54.4^1^	61.4^1^
**Chinese**	17.9	31.9	30.7	21.7
**Indian**	9.5	8.3	6.5	6.5
**Orang Asli**	2.2	1.3	na^1^	na^1^
**Other**	2.4	0.3	0.5	0.9
**Non-citizens**	5.6	5.6	8.0	9.6

SEACO estimates were according to the baseline enumeration, conducted in 2012–2013.

District-level estimates are reported from the Department of Statistics, Malaysia (2010).

State and national estimates are reported from the Department of Statistics, Malaysia (2013).

^1^Estimates include Orang Asli and Malay as one ethnicity (Bumiputera)

At the primary care level, the Malaysian health system comprises community nurse clinics (Klinik Desa, or KD) and health clinics (Klinik Kesihatan, or KK). KDs provide basic antenatal, postnatal, vaccination and family planning services, whereas KKs provide a wider range of medical and dental services. Segamat district as a whole meets the government requirement of four KDs and one KK per 20 000 individuals.^3^^,^^7^ The major public tertiary care centre in the area is the Segamat District Hospital, located in Sungai Segamat, where a number of private medical and dental clinics also operate.

## Who is included and how often are they surveyed?

### Coverage of participants during enumerations, surveys and studies

SEACO undertook a baseline enumeration over 2012–13, and conducts annual enumeration updates within its five sub-districts; two updates have been completed since the baseline enumeration. All individuals living within the SEACO sub-districts are approached each year for participation in the enumeration and updates. These are conducted using a census approach by local data collectors, and are addressed to heads of household. If no inhabitants are present during the initial visit to a household, it is re-visited a maximum of three times within the data collection period, after which it is listed as a non-responding household until the next enumeration update. Households declining to participate are also approached at a later date and, with appropriate consent, are asked about reasons for which they chose not to participate. Consenting households are tagged with an adhesive barcoded card to enable linkage to individuals in the home. In total, 44 902 individuals living in 13 355 households were covered in the baseline enumeration, accounting for approximately 85% of the total population estimated to be living within the area. This level of participation has been maintained in all following enumeration updates.

Households where a death is reported to have taken place are visited later, at an agreed convenient and culturally appropriate time, for verbal autopsies to determine the cause of death. Births are identified through the KKs, mothers are visited within 3 months of delivery and the infant is enrolled. In addition, antenatal, delivery and birth data can be linked through the electronic records compiled by the National Obstetrics Registry at the Segamat District Hospital.

The SEACO infrastructure supports multiple research projects additional to the annual enumeration updates. This includes a population-wide health survey undertaken in 2013–14, conducted at the individual level among participants aged 5 years and above. Recruiting 25 184 participants (a little over 50% of the estimated total population), this survey aimed to provide a baseline view of the overall health of the population, including medical history, mental well-being, non-communicable disease risk factor status and health service use. Further focused, smaller-scale studies based on subsets of the local population are currently ongoing; these span a wide range of research disciplines and topics, from the prevalence of dengue to household expenditures related to diabetes. Eligible participants for these studies are identified through community informants, or through enumeration updates, during which consent for later re-approach is also sought. The specific nature of these studies determines the frequency with which participants are followed up.

### Linkage and tracking of HDSS participants

All individuals participating in SEACO-led research are assigned a unique SEACO identifier (ID) upon first contact. This ID is used during all following contact with SEACO, allowing for any individual’s health and demographic information to be linked across enumeration data, surveys and studies and tracked longitudinally. In addition, all participants who are Malaysian citizens possess a National Registration Identification Card (NRIC), a record of which is taken by SEACO.^8^ This allows for confirmation of correct identification of a participant during later enumeration updates, surveys or studies. The NRIC is also used in public medical facilities, giving SEACO the potential capacity to link individuals’ information to their clinical data. The regularity and linked nature of follow-up in the population covered by SEACO thus allows for the potential to build a comprehensive, longitudinal picture of the health status of individuals covered by the HDSS.

## What has been measured and how have the HDSS databases been constructed?

### Information collected during enumerations, surveys and studies

The annual enumeration updates involve regular collection and updating of demographic indicators. Information collected during enumeration updates includes: (i) questions asked at baseline (initially asked during the baseline enumeration, and currently asked of each newly-immigrated household within the area); (ii) a core set of questions repeated each year; and (iii) an extended set of questions distinct to each year. Questions asked at baseline include basic information such as dwelling construction materials, or the number of household members. Core questions include those directed towards recording in-migrations, out-migrations, births and deaths. The extended questions capture various aspects of socioeconomic, demographic or health status, or aim to identify individuals or households of interest for later follow-up in more focused studies. They have thus included queries ranging from monthly household expenditures and assets to self-reported incident cases of stroke among household members (Table 2).

**Table 2:** Information collected during enumeration and enumeration updates in the SEACO HDSS

Item	Information	Baseline^1^	Core^2^	Extended^3^
Homestead	GPS location	x	x	
	House address; confirmation of house address	x	x	
	House type (e.g. bungalow), construction material, nature of ownership (e.g. rented)	x		
	Number of bedrooms, bathrooms, living areas other than kitchen	x		
	Main source of drinking water; main method of garbage disposal			x
	Type of toilet; whether shared			x
Assets	Number of cars owned; number of motorcycles owned	x		
	Availability of internet and television	x		
Expenditures	Expenditures on food and drink items, alcohol and tobacco			x
	Expenditures on housing and fuel, health, education and taxes, other household goods			x
	Expenditures on family and religious commitments e.g. religious ceremonies			x
Household socioeconomic status	Sufficiency of household earnings to meet overall, basic nutritional, and healthcare needs			x
	Self-perception of socioeconomic position compared with other households			x
	Household monthly income category			x
	Homestead condition (e.g. repairs needed)			x
	Major renovations, room additions or repairs (e.g. re-wired, new plumbing)			x
	Capacity to collect RM^4^ 1500 for an emergency in 24 hours	x	x	
Household	Family type (e.g. nuclear or extended)	x		
	Number of members, number living in the household for greater than 3 months	x		
Household total outmigration	Reasons why house is empty		x	
	Where household moved to; exact address		x	
Outmigration	Reasons for no longer being part of the household		x	
	Where individual moved to; exact address		x	
Inmigration	Reasons for moving into household		x	
	Identity document and ID number		x	
Deaths	Date died or time since death		x	
	Consent for later follow up in verbal autopsies		x	
Births	Mother's and father's full names		x	
	Date of birth		x	
	Location of birth			x
	Relationship to head of household			x
	Expectant mothers; expected date of delivery; expected location of delivery		x	
Individual residents’ characteristics	Identity document and ID number	x	x	
	Name, date of birth, age at last birthday, sex, citizenship, ethnicity, religion, marital status, main language	x	x	
	Relationship to head of household	x	x	
	Schooling history, employment status, job title and classification	x	x	

^1^Baseline: asked during the baseline enumeration and to newly-immigrated households; ^2^Core: repeated annually;^ 3^Extended: distinct to each year; ^4^RM: Malaysian Ringgit.

The verbal autopsy register containing a list of deaths within the community is compiled using information obtained from the enumeration updates, and from updates throughout the year from key community informants. Households in which deaths have occurred in the past year are approached with consent at later, culturally appropriate and convenient dates, for verbal autopsies. These are performed using the World Health Organization 2012 verbal autopsy instrument, adapted according to the local context in consultation with the Ministry of Health.^9^

Conducted population-wide over 2013–14, the health survey is the other major data collection project undertaken within the SEACO population. This survey was directed towards capturing participants’ medical history, behavioural and biophysical risk measures for non-communicable diseases, and indices of well-being. Information in this survey was collected using methods adapted from standardized health data collection tools, including the World Health Organization (WHO) STEPwise approach to Surveillance (STEPS, for non-communicable disease risk factor surveillance),^10^^,^^11^ WHO Study on global AGEing and adult health (SAGE),^12^ WHO Quality of Life (QOL)^13^ and Depression Anxiety and Stress Scales (DASS)^14^ (Table 3).

**Table 3:** Selected measures collected during the health survey in the SEACO HDSS

Item	Information
Demographic and socieconomic characteristics	Identity document and ID number
	Name, date of birth, age at last birthday, sex, citizenship, ethnicity, marital status
	Relationship to head of household
	Literacy, schooling history, employment in past 30 days
	Gross monthly income from own work, other household members, and members outside of household
WHO STEPwise approach to Surveillance - adapted	Medical history: heart disease, kidney disease, asthma, dengue fever
	Experience of health problems such as troubled breathing or vomiting, stomach pain or dizziness in past two weeks
	Experience of healthcare seeking for recent health problems (consultation, medicine and transportation time and costs)
	Frequency of intake of any traditional medicine
	Behavioural risk factors for non-communicable diseases: smoking, alcohol, nutrition, physical activity
	History of, and use of medicine for, hypertension or diabetes
	Biophysical measures: height, weight, waist circumference, blood pressure, random blood glucose
Dental health	State of teeth; frequency and intensity of oral discomfort
	Most recent dental examination, date and location
Depression Anxiety Stress Scales (DASS-21)	Experience of symptoms of depression, anxiety and stress within the past week
WHO Quality of Life (WHO QOL- BREF)	Self-perception of health status, enjoyment of and satisfaction with life course, living conditions and interpersonal relationships
WHO Study on global AGEing and adult health (SAGE) - adapted	Difficulty in functioning in the past 30 days (e.g. difficulty sleeping, or learning a new task)
	Recent trips or falls
	Attachment to local community, active involvement in community social activities or clubs
	Social connectedness: daily contact with others, use of a smartphone or tablet, or use of email or social media

Whereas data from the health survey allow for a broad understanding of population-wide measures of disease risk or well-being, multiple studies embedded within the SEACO infrastructure also collect detailed data on specific diseases and conditions of interest to the community. Often multidisciplinary in nature, these studies have generated in-depth evidence on a range of key health topics, including the prevalence of dengue infection within the community, sexual health in the elderly, post-stroke recovery and rehabilitation, the economic burden of diabetes, health-promoting behaviours in youth and cognitive decline in the elderly (Table 4).

**Table 4:** Selected research projects currently on-going in the SEACO HDSS

Theme	Project
**Communicable disease**	Prevalence and associated risk factors for dengue infection in Segamat (immunology, entomology, genetics, clinical care)
**Non-communicable disease**	The economic burden of diabetes care (health economics, medical anthropology)
**Non-communicable disease; adolescent health**	Qualitative understanding of health behaviours in adolescents' transition to adulthood (medical anthropology)
	Ethnographic exploration of youths' dietary practices in Segamat (medical anthropology)
	Household level influences on risk factors for non-communicable disease among children in Segamat (epidemiology)
**Health in the ageing population**	Cognitive decline in the elderly (epidemiology)
	Health and social care of elderly singletons (medical anthropology)
	Healthcare of the chronically ill elderly: coping measures and outcomes (medical anthropology)
**Stroke**	Recovery and well-being following stroke (epidemiology, medical anthropology)
	The effect of coping strategies and social support on recovery following stroke (medical anthropology)
	Potential disabling effects of informal care giving on stroke caregivers (medical anthropology)

### Data collection and database management procedures

All raw data are collected using electronic questionnaires on encrypted android tablets. Questionnaires, including appropriate built-in checks for data entries, are initially designed on a spreadsheet. The spreadsheet is converted to XML format, and then interpreted into questionnaire form on the android tablets by Open Data Kit Collect, an open-source mobile application.^15^ For the enumeration and updates and the health survey, additional data collection checks include random repeat visits of households or accompaniment of data collectors by field supervisors and field coordinators. Data are uploaded once a week from android tablets to a secure server, and the completeness and validity of data entries are checked by the database management team. Data collectors revisit households or individuals about whom collected data are incomplete or ambiguous. All data are linked-anonymized, and are held on secure study servers at Monash University Malaysia.

## Key findings and publications

**Figure 2:**
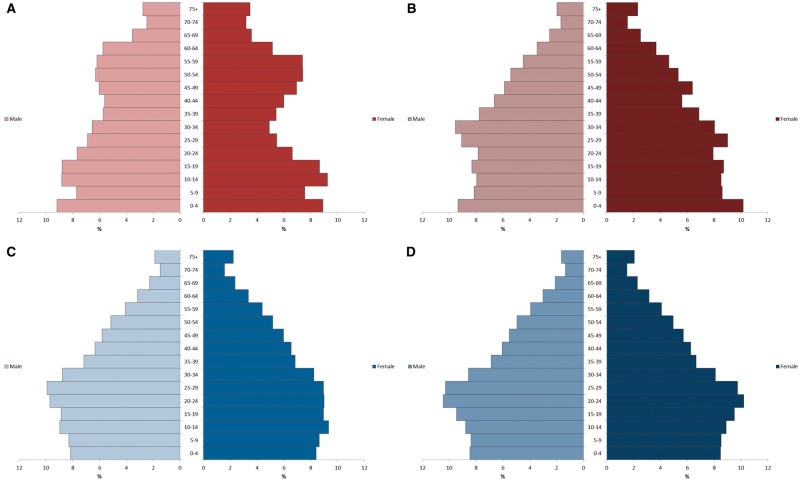
Population pyramids for (A) SEACO (2012–2013), (B) Segamat district (2016), (C) Johor state (2013) and (D) Malaysia (2013). Estimates from SEACO are based on the baseline enumeration. District, state and national estimates are based on reports by the Department of Statistics, Malaysia.

As reported in a recent publication, the age structure of the SEACO population is notably different from population profiles based on district, state and national data, with a smaller proportion of young and middle-aged adults, and a greater proportion of older adults.^16^ In population pyramids generated from SEACO sub-districts, the proportion of individuals aged 20–45 years was lower than corresponding proportions in district, state and national population pyramids. This may be a result of outmigration of working-age individuals seeking better education or employment opportunities (Figure 2). The dependency ratio for older dependants was estimated to be 0.15, close to double the national estimate of 0.08; the dependency ratio for younger dependants was comparable but marginally lower than national estimates (SEACO: 0.37, national: 0.45). This has implications regarding the nature of the expected disease burden, and therefore the unique health service needs, of this and other rural populations.^16^

Other SEACO-based publications to date relate to methodological issues surrounding the establishment and operation of the HDSS.^3^^,^^9^^,^^16–19^ These publications are important in the context of the paucity of literature surrounding such topics, including the effective navigation of local cultural and religious customs during study planning and data collection. Relevant publications have included a case study of community engagement methods employed in Segamat before undertaking the baseline enumeration.^3^ This was a fundamental exercise directed at encouraging participation, given that there was no demonstrated need for functions that other HDSS sites typically perform, such as vital registration. Another case study has discussed the operational aspects of conducting verbal autopsies within the multi-ethnic and multi-faith community covered by SEACO.^9^ Finally, a recent study used data from the SEACO baseline enumeration to demonstrate the potential effectiveness of a Bayesian method to predict the ethnicity of individuals, given their names.^17^ A list of publications arising from the SEACO HDSS can be found at [http://www.seaco.asia/research/publication/].

## Future analysis plans

A number of multidisciplinary projects using the SEACO infrastructure are currently at various stages of completion. A subset of these projects is currently analysing enumeration and health survey data, to enhance understanding of the socio-demographic determinants and consequences of non-communicable diseases and their risk factors. Other ongoing analyses are tied to specific studies, as discussed previously and presented in Table 4.

Preparations for future large-scale data collections using the SEACO framework are currently under way. These projects are expected to greatly broaden the scope of measures currently taken, through the addition of comprehensive biomarker data. Planned projects include a second population-wide health survey which will additionally incorporate the collection of blood and other biological samples. Furthermore, a feasibility study is under way for the establishment of a birth cohort, with the collection of key health information and biological samples from women and their offspring throughout pregnancy and after birth. Given the relative scarcity of large-scale health surveys or birth cohorts based in Asia, these planned data collections are expected to fill an important gap in our knowledge of the relative effects of genetic and environmental influences on the development of health and disease in Asian populations. A further feasibility study is exploring similar questions for an ageing cohort.

A comprehensive list of planned publications and ongoing SEACO-based research projects can be found at [http://www.seaco.asia/research/publications-work-in-progress/ and http://www.seaco.asia/research/seaco-research-project/].

## Strengths and weaknesses

One of SEACO’s key strengths is its coverage of a large population in Malaysia. Based in a multi-ethnic middle-income country, it is well-poised and well-powered to reliably assess the influence of a wide range of biological and environmental exposures on health and disease. Furthermore, the collection of individuals’ National Registration Identification (NRIC) information provides a valuable opportunity for participants’ measures to be linked to clinical data stored in public medical facilities. It is expected that this linkage will provide a more detailed view of participants’ health trajectories, along with greater opportunities for prospective ascertainment of diseases of interest, in future studies.

Engagement with the community and partnership with the local government, the Ministry of Health and other relevant stakeholders, are the cornerstone of SEACO’s functioning and purpose. The HDSS has a strong community engagement programme, with regular community-wide events and local recruitment of field staff. SEACO’s sub-district-specific community engagement committees (CECs) comprise key community leaders with representation from various community stakeholder groups who guide research priorities, provide vital input on operational issues relevant to the undertaking of research projects and play an important role in building trust within the community. The local government facilitates access to households and arrangements for health care referrals for individuals identified to be potentially at risk according to survey measures.

SEACO is the only health and demographic surveillance system to have attained the International Standards Organization (ISO) certification for quality management (ISO 9001:2008), and is an associate member of the International Network for the Demographic Evaluation of Populations and their Health (INDEPTH network). With appropriate and rigorous training of all field staff, the HDSS has an important function in building capacity and health literacy in the community, and is capable of providing programmatic support to both qualitative and quantitative studies based within its community.

Operationally, SEACO has faced challenges with retaining local data collectors, due to the seasonal nature of the work. As a result, the pace of data collection for large surveys can vary considerably, and new data collectors have to be constantly recruited and trained. Correct identification of participants during follow-up has been another operational challenge, since individuals in the community often share identical names, or interchangeably use formal and informal or differently-spelled names. Although the use of the NRIC could solve this, some participants have expressed unwillingness to repeatedly share such identifying details. A potential solution to this, currently being explored, is the issue of barcoded SEACO cards to each individual participant, linked to their SEACO IDs. This is expected to greatly improve the efficiency and accuracy of participant identification and linkage of information. As can be expected of low-level administrative units, the population covered by SEACO is demographically distinct compared with the district, state and national population (Table 1, Figure 2). As such, SEACO is not intended to generate nationally representative findings, but rather provides the opportunity to undertake large-scale epidemiological analyses and in-depth nested studies.

## Data sharing and collaboration

SEACO welcomes opportunities for data sharing and collaboration. Data are held in secure servers at Monash University. Data dictionaries for the enumeration and updates, the health survey and other SEACO studies can be accessed at [http://www.seaco.asia/codebook/]. Instructions on applying for the release of data for research purposes, and guidelines on applying for collaborations on research projects, can be found at [http://www.seaco.asia/research/how-to-collaborate-with-seaco/]. Any queries regarding potential collaborations can be directed to Daniel D Reidpath (Director, SEACO) at [daniel.reidpath@monash.edu].

**The SEACO HDSS Profile in a nutshell**

- The SEACO HDSS covers five of 11 sub-districts of Segamat district in southern peninsular Malaysia, a total area of approximately 1250 km^2^. SEACO is unique in its location in a multi-ethnic country undergoing economic, demographic and epidemiological transitions; this backdrop allows the HDSS to capture a wide range of factors that influence health and disease in this region.
- A total of 44 902 individuals living in 13 355 households was covered during the baseline enumeration, conducted over 2012–13.
- Enumeration updates are undertaken annually and include questions on individual and household socio-demographic indices and health measures of potential relevance to other SEACO-based studies. Registration of newborns and verbal autopsies to determine cause of death are performed throughout the year.
- SEACO has additionally undertaken a health survey (2013–14) which collected information ranging from behavioural and biophysical risk measures for non-communicable disease to indices of well-being. It also provides the infrastructure for multiple focused studies on health issues of interest to the community, such as dengue infection or post-stroke recovery and rehabilitation.
- SEACO welcomes invitations for data sharing and collaborations [http://www.seaco.asia/research/how-to-collaborate-with-seaco/]. Requests for further information on opportunities for data sharing and collaboration can be directed to Daniel D Reidpath [daniel.reidpath@monash.edu].

##  Funding

SEACO is funded by the office of the Vice Provost Research, Monash University Australia; the office of the Deputy Dean Research, Faculty of Medicine, Nursing and Health Sciences, Monash University Australia; the Monash University Malaysia Campus; and the Jeffrey Cheah School of Medicine and Health Sciences. SEACO is an associate member of the INDEPTH Network. This work was supported by the Wellcome Trust (grant number 098051). M.S. is supported by the National Institute for Health Research Cambridge Biomedical Research Centre (UK). U.P. is supported by the Dr Herchel Smith Fellowship.
